# Direct Fibrinolytic Snake Venom Metalloproteinases Affecting Hemostasis: Structural, Biochemical Features and Therapeutic Potential

**DOI:** 10.3390/toxins9120392

**Published:** 2017-12-05

**Authors:** Eladio F. Sanchez, Renzo J. Flores-Ortiz, Valeria G. Alvarenga, Johannes A. Eble

**Affiliations:** 1 Research and Development Center, Ezequiel Dias Foundation, Belo Horizonte 30510-010, MG, Brazil; eladio.flores@funed.mg.gov.br; 2 Graduate Program in Nursing, Federal University of Minas Gerais, Belo Horizonte 30130-100, MG, Brazil; renzojfo@gmail.com; 3 Institute for Physiological Chemistry and Pathobiochemistry, University of Münster, 15, 48149 Muenster, Germany; johannes.eble@uni-muenster.de

**Keywords:** metalloproteinases, animal toxins, thrombolysis, antithrombotics

## Abstract

Snake venom metalloproteinases (SVMPs) are predominant in viperid venoms, which provoke hemorrhage and affect hemostasis and thrombosis. P-I class enzymes consist only of a single metalloproteinase domain. Despite sharing high sequence homology, only some of them induce hemorrhage. They have direct fibrin(ogen)olytic activity. Their main biological substrate is fibrin(ogen), whose Aα-chain is degraded rapidly and independently of activation of plasminogen. It is important to understand their biochemical and physiological mechanisms, as well as their applications, to study the etiology of some human diseases and to identify sites of potential intervention. As compared to all current antiplatelet therapies to treat cardiovascular events, the SVMPs have outstanding biochemical attributes: (a) they are insensitive to plasma serine proteinase inhibitors; (b) they have the potential to avoid bleeding risk; (c) mechanistically, they are inactivated/cleared by α2-macroglobulin that limits their range of action in circulation; and (d) few of them also impair platelet aggregation that represent an important target for therapeutic intervention. This review will briefly highlight the structure–function relationships of these few direct-acting fibrinolytic agents, including, barnettlysin-I, isolated from *Bothrops barnetti* venom, that could be considered as potential agent to treat major thrombotic disorders. Some of their pharmacological advantages are compared with plasmin.

## 1. Introduction

Among the venomous animals, snakes are the best-studied creatures throughout human history; this is partially due to the bad reputation associated with snakes, as many people have experienced that these small and often fragile-looking animals are harmful to man, and can inflict devastating damage in envenomed victims [1]. Indeed, snake venoms, especially those of the Viperidae (pit vipers and true vipers) family, contain extremely complex mixtures of pharmacologically active proteins/peptides that disrupt normal physiological or biochemical processes in line with their function to immobilize, to kill, and to digest their prey, as well as to defend themselves from predators [2,3]. They belong to a few structural classes of major protein families, including proteins with and without enzymatic activity, such as metalloproteinases (SVMPs), serine proteinases (SVSPs), phospholipases A_2_ (PLA_2_s), l-amino acid oxidases (l-AAOs), hyaluronidases, and non-enzymatic proteins: disintegrins, C-type lectin-like proteins/snaclecs, bradykinin-potentiating peptides (BPPs), nerve and vascular endothelial grow factors (VEGF), and Kunitz-type proteinase inhibitors [2,3,4,5]. Acting synergistically, various venom proteins/toxins are able to cause severe and detrimental effects on the hemostatic system and lead to cardiovascular shock [5,6,7]. Moreover, several of these compounds offer interesting and often unique insights into several biological systems [7,8,9]. In the context of drug discovery, the isolation and characterization of active venom proteins/toxins is carried out for two main purposes: (1) to identify and determine the compound(s) responsible for a specific activity observed in a bioassay; or (2) to survey the complete structural diversity of a venom to discover new sequences with novel structural scaffolds and pharmacological properties. Therefore, after a snake venom constituent has been properly purified, after its molecular structure has been resolved and its specific pharmacological effects have been revealed, the resulting pharmaceutical lead structure and the known molecular mechanism are beneficial to mankind, in contrast to the envenomation with the crude venom [3,7,8,9,10]. Underpinning research in the biomedicine field, such as looking for new thrombolytic and/or antithrombotic agents, becomes of increasing medical importance. It is noteworthy that around half of the drugs which are currently in therapeutic use have originated from natural products [10,11].

Snake venom metalloproteinases (SVMPs) are the crucial endopeptidases associated with the pathologies of snake envenoming. Especially coagulopathies commonly associated with viperid (*Serpentes viperidae*) are caused by enzymatic and non-enzymatic proteins in these venoms, and usually lead to incoagulability of the blood [12,13,14]. Proteomic analysis of snake venoms showed that in some venoms they are the most abundant >50% proteins of the proteome, e.g., see reference [15], and most lethal protein in viper and pit viper venoms [13,14,15], but are less significant in the venoms of Elapidae, Atractaspididae and Colubridae. In addition, differential proteomic compositions among different families of snakes have been reported recently [16]. SVMPs represent a group of multigene protein families that encode different multidomain protein molecules capable of producing a diverse array of activities, including hemorrhage, pro-coagulant, anticoagulant, fibrinolysis, apoptosis, and antiplatelet effects [1,15,16]. They were inferred to be derived through recruitment, duplication, and neofunctionalization of an ancestral gene, which must have been very similar to the recent genes encoding ADAM7, 28, and ADAMDEC-1 [1]. Consequently, SVMPs/reprolysins are also referred to as snake ADAMs (a disintegrin and metalloprotease). Actually, the large P-III class metalloproteases have a modular structure that is homologous to the ectodomain of membrane-anchored ADAMs [17,18]. Furthermore, SVMPs, ADAMs and ADAMTSs, the latter of which contain an additional thrombospondin (TS) motifs, share a topological similarity to matrix metalloproteinases of tissues (MMPs) within the structural organization of their catalytic center [19]. However, their non-catalytic ancillary domains are distinct from those of MMPs and other metalloproteinases [20]. On the other hand, SVMPs selectively hydrolyze a small number of proteins involved in key reactions in the coagulation cascade, fibrinolysis, and in platelet function. Such mechanism of action leads to either activation or inactivation of the protein that participate in these processes, thereby severely interfering with blood coagulation and platelet function. In the following sections, we provide an overview of several direct-acting fibrinolytic P-I metalloproteinases that affect hemostasis and impair platelet function. Their potential application in therapy of major arterial occlusive disorders is surveyed.

## 2. Structure and Classification of Snake Venom Metalloproteinases

**Figure 1:**
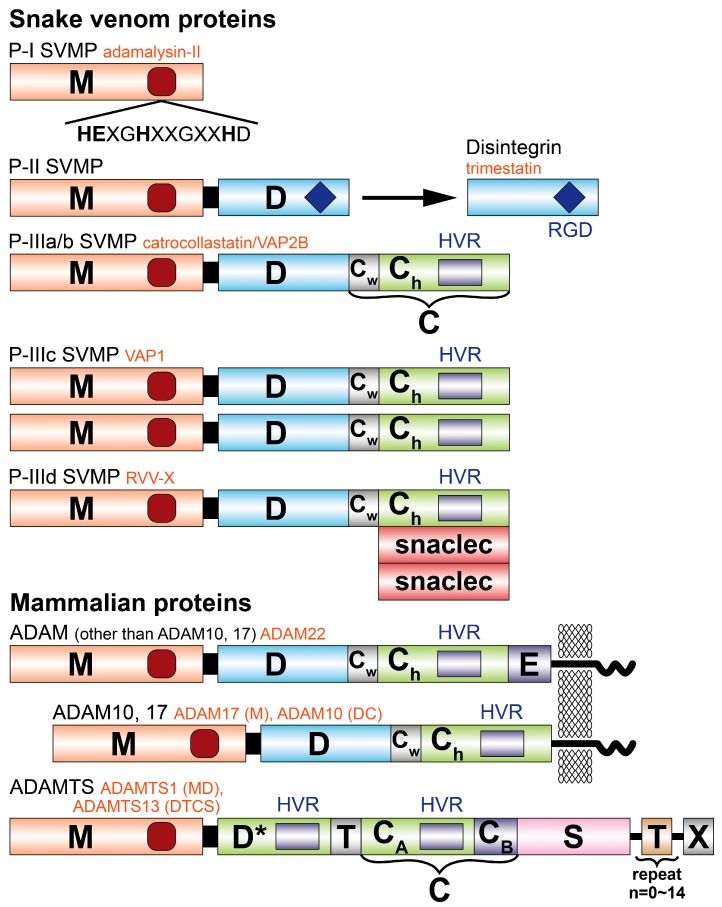
Protein domain structure of snake venom metalloproteinases (SVMPs) and related molecules. Each domain or subdomain is represented by a different color. M, metalloproteinase; D, disintegrin (or disintegrin-like) domain; C, cysteine-rich domain; C_W_, cysteine-rich “wrist” subdomain; C_h_, the cysteine-rich “hand” subdomain; snaclec, snake venom C-type lectin-like domain; E, epidermal growth factor (EGF)-like domain; T, thrombospondin type-1 (TSP) motif; S, spacer domain; X, domain variable among ADAMTSs. Representatives of each class of SVMPs and ADAM/ADAMTSs, whose crystal structure have been determined, are indicated in red letters. The P-III classes SVMPs are divided into subclasses (IIIa–IIId) based on their distinct post-translation modifications. Recently, it was found that the D domain of ADAMTS family proteinases does not have a disintegrin-like structure but adopt the C_h_ subdomain fold, and thus, is represented as D*. The previously cysteine-rich domain of ADAMTSs is structurally subdivided into the N-terminal G_h_ subdomain-fold domain (C_A_) and the C-terminal domain (C_B_). The ADAMTS family commonly possesses the N-terminal M, D, T, C, S domains whereas the C-terminal is variable among ADAMTSs e.g., ADAMTS13 possess six repeats of TSP and two CUB (complement, uEGF, and bone morphogenesis) domains that follow the S domain. Reproduced from [14], copyright 2012, Elsevier.

SVMPs are zinc-dependent endopeptidases also known as adamalysins/reprolysins, based on the name of its structural prototype adamalysin II, isolated from eastern diamondback rattlesnake (*Crotalus adamanteus)* venom, and on the mammalian reproductive tract proteins involved in cellular adhesion [21,22,23]. These enzymes are also termed as ADAMs (a disintegrin and metallropteinase), MDC (metalloproteinase-like, disintegrin-like and cysteine-rich proteins), and are grouped into three major classes, P-I to P-III, according to their general structural organization, and are subdivided into several subgroups (Figure 1) [19,24,25]. They were initially characterized by their ability or inability to induce hemorrhage in experimental in vivo models [26,27]. Hemorrhage is defined as the escape of blood from the vascular system. This leaking is caused by damage of the vessel wall, which consists of the endothelial cell layer and the subjacent extracellular matrix, such as basement membranes and interstitial stroma. Proteolytic cleavage of extracellular matrix proteins, of blood clotting factors, and of cell adhesion receptors on platelets and endothelial cells by SVMPs are the main reason for venom-induced hemorrhages.

**Figure 2:**
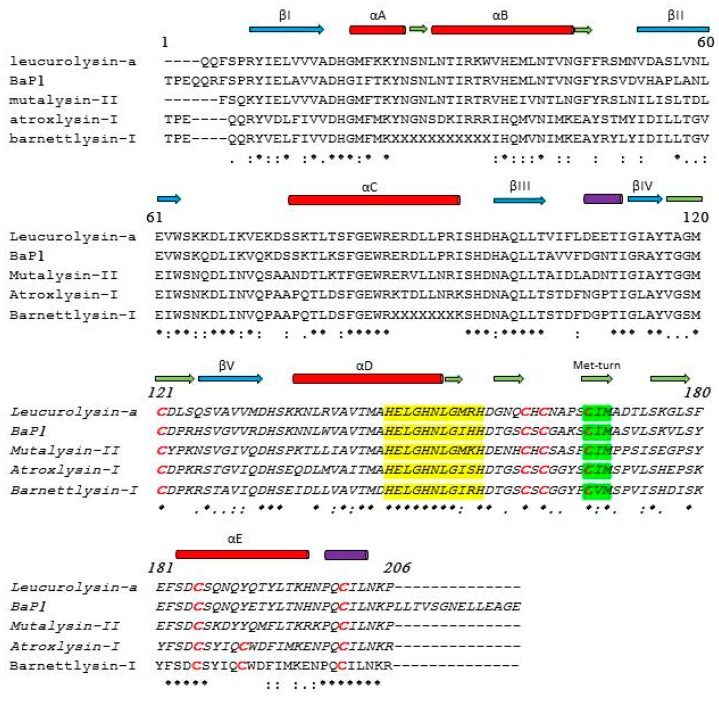
Sequence comparisons of four P-I class SVMPs. UniProt accession numbers sequences were assigned by using the program ClustalW. Non-hemorrhagic: leuc-a (P84907), mut-II (P22796), bar-I (P86976), and hemorrhagic: atr-I (P85420) and BaP1 (P83512). The sequences of these proteins were determined by the Edman degradation method and the sequences of leuc-a and BaP1 were confirmed by crystallography. Secondary-structure elements were defined by MAFFT V7 (multiple alignment) and PSIPRED V3.3 (predict secondary structure). The blue and dark green arrows indicate the locations of β-strands and turns, respectively, in the crystal structure of leuc-a. The red and purple cylinders represent α-helices and 3_10_ helices, respectively. Cys residues are highlighted in red; (*) identical residues; (:) strongly similar residues; (.) weakly similar residues. The conserved zinc biding motif and the met-turn are highlighted in yellow and bright green, respectively. (-) indicate gaps.

Class I (P-I) SVMPs, have a single catalytic metalloproteinase (MP) domain in their mature form [23,28,29,30]. All SVMPs exhibit an extended zinc-binding consensus sequence HEXXHXXGXXH/D, which comprises three zinc-coordinating histidine side chains, and generally, a glutamate residue. Moreover, these proteins also possess a strictly conserved methionine containing 1,4-β-turn, termed Met-turn, bordering the substrate-binding site, which is a typical feature of the metzincin clan of metalloproteinases [19,21,31]. In general, there are two structural forms of the proteinase domain: a two-disulfide-containing structure e.g., in adamalysin II [19,21] and a three-disulfide-stabilized structure e.g., in mutalysin-II (mut-II) [30,32] and in leucurolysin-a (leuc-a) [29]. Sequence alignment of the P-I enzymes indicate that they possess high sequence homologies (Figure 2).

Based on the functional ability to induce hemorrhage, the P-I SVMPs are further divided into two subgroups: P-IA which induce hemorrhage [28,33], and P-IB with weak (or no) hemorrhagic effect [29,32,34]. SVMPs play important roles in the overall pathophysiology of viperid envenoming by inducing local and systemic hemorrhage, which was primarily attributed to their potential to degrade basement membrane (BM) components surrounding capillaries, like type IV collagen, laminin (LM), nidogen, and fibronectin (FN), as well as to induce other tissue damaging and hemostatic alterations [8,22,25,35,36,37].

In addition to the MP domain, class II (P-II) SVMPs possess a C-terminal disintegrin domain (Dis). A group of disintegrins released from precursor P-II MPs have an RGD motif, which mediates the interaction with integrins, thereby offering many potentials for pharmacological applications [7,38,39]. Other active tripeptide sequences such as KGD, MDV, MLD, VGD, ECD, MDG, and KTS have been reported [40]. The RGD and KGD tripeptide sequences are the primary recognition sites for the integrin αIIbβ3 receptor. The binding of disintegrin to integrin αIIbβ3, blocks the binding of fibrinogen to the receptor, and hence, platelet aggregation [7,26,39,40]. Two FDA-approved drugs, Eptifibatide (Integrilin^®^, Millennium Pharmaceuticals, Shering-Plough, Cambridge, MA, USA), and Tirofiban (Aggrastat^®^, Merck, Darmstadt, Hesse, Germany), antagonists of the platelet receptor glycoprotein αIIbβ3 of human platelets, inhibit platelet aggregation, and are the first rationally designed antiplatelet agents [9,41,42].

Class III (P-III) SVMPs contain the MP, disintegrin-like (containing a disulfide-linked XXCD, mostly SECD, in place of RGD) and cysteine rich (Cys) domains, and are the most mysterious enzymes in terms of complexity and function. Their structure is homologous to a group of membrane bound ADAM, which act in cell–cell and cell–matrix adhesion and signaling [14,24]. P-III SVMPs are further grouped into subclasses based on their different post-translational modifications, such as homo-dimerization (P-IIIc), proteolysis between the MP and Dis domain (P-IIIb), or complexation (P-IIId) with additional snake C-type lectin-like proteins (snaclecs) [43]. All SVMPs have a signal sequence (pre-form) and a zymogenic sequence (pro-form) N-terminal to the MP domain in their gene structures. The signal sequence is cleaved co-translationally in the endoplasmic reticulum, whereas cleavage of the zymogenic sequence occurs extracellularly, is regulated, and activates the proteolytic enzyme. Evolution of viperid SVMPs is characterized by domain loss along the evolutionary timeline, thus, the loss of the Cys domain had preceded the development of the P-II class, which in turn preceded the formation of the P-I SVMPs [17,44].

## 3. Three-Dimensional Structures of P-I Class SVMPs

**Figure 3:**
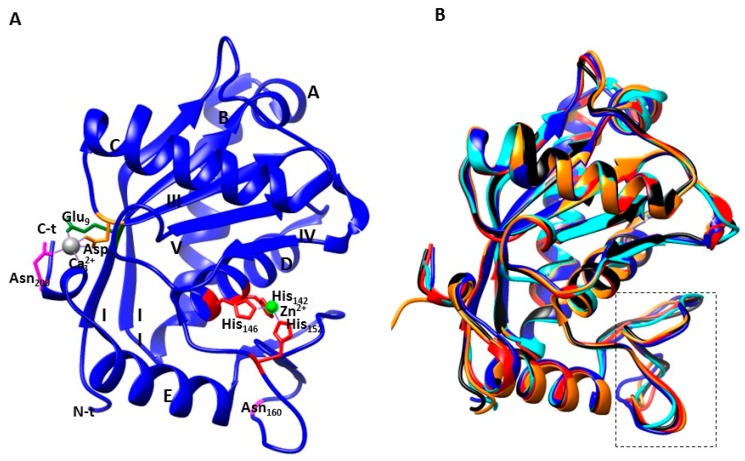
Ribbon plot of the overall structure of metalloproteinase leucurolysin-a (**A**) and its superposition with the structures of other P-I SVMPs (**B**), depicted in standard orientation. (**A**) The molecular structure of leucurolysin-a, a non-hemorrhagic P-I SVMP. The catalytic zinc atom is highlighted in green, together with the three histidines (red), a glutamic acid, and the water molecule forming the active site environment. The localization of α-helices (A–E), β-strands (I–V) and the methionine-turn as well as the N- and C-terminal residues are also indicated; (**B**) Superposition of structures by UCSF CHIMERA system of non-hemorrhagic P-I SVMPs: leuc-a (blue, PDB 4Q1L; mut-II, red, P22796, predicted by I-Tasser program C-score 1.58; bar-I, cyano, P86976, predicted by I-Tasser program C-score 1.49), and hemorrhagic P-I SVMPs: BaP1 (orange, PBD 2W12), and atr-I (black, P85420, predicted by I-Tasser program C-score 1.49). The superposed structures within the Met-turn are highlighted in the insert figure. However, flexibility of this variable motif does not provide relevant details responsible for hemorrhagic activity.

Table 1, summarizes the three-dimensional structures currently available for eleven P-I class SVMPs, as well as their main proteolytic activity related to hemorrhage. The molecular structure of P-I SVMP adamalysin II from the eastern diamondback rattlesnake (*Crotalus adamanteus*) venom was the first one of the M12B proteinase to be solved by X-ray crystallography in 1993 [21]. The first P-III SVMP, vascular-apoptosis inducing protein-1 (VAP-1) was reported by 2006 [18]. The 3D structures of a number of P-I SVMPs soon followed, and to date, the structures of eleven P-I proteinases are available in the Protein Data Bank (PDB) [20]. By the 1990s, the Sanchez lab (Biochemistry of Proteins from Animal Venoms) at FUNED, Brazil, started to investigate/identify fibrinolytic activity of the small SVMPs from bushmaster snake (*Lachesis muta muta*). Later, this research was extended to other South American *Bothrops* snakes. Thus, other P-I class enzymes, including leucurolysin-a (leuc-a) from the venom of the Brazilian snake *Bothrops leucurus* (white-tailed jararaca), were discovered and described [29]. The mature leuc-a is composed of 202 amino acid residues, and was crystallized using the hanging-drop vapor-diffusion technique at 1.8 Å. The crystal structure of leuc-a (PDB code 4Q1L) complexed with an endogenous tripeptide (QSW) was solved by molecular replacement technique using the proteinase BaP1 (*B. asper*) structure (PDB code INDI) as template Ferreira et al., unpublished [45]. The crystal structure analysis reveals that leuc-a is an ellipsoidal molecule with a relatively flat active-site cleft that separates two subdomains similar to the two jaws of the oral cavity (Figure 3). The upper jaw is formed by the N-terminal subdomain of the molecule (residues 1-152) and characterized by a β-strand with four parallel and one antiparallel β-strand (strands I, II, III, IV, and V), which is flanked by a long and short surface located helix on its convex side, and by two long helices, one of which represents the central active site helix, on its concave side. The lower jaw, comprising the 50 C-terminal residues, is folded in a more irregular fold, which is organized in multiple turns, with the chain ending in a long C-terminal helix and an extended segment that is linked to the upper subdomain by a disulfide bond. The catalytic zinc ion is located at the active site cleft between the two subdomains (jaws). It is tetrahedrally coordinated by His^142^, and His^146^ of the upper subdomain, by His^152^ of the lower subdomain, and a water molecule, which is polarized by Glu^143^, and therefore attacks the scissile peptide bond in a nucleophilic manner. These three His residues and the nearby Glu play a critical role in both the structure and activity of P-I proteinases, and explains their occurrence in the H^142^EXXHXXGXXH^152^D consensus sequence. In addition, Asp^153^ is strictly conserved in the SVMPs that establish a hydrogen bond with an invariant serine (Ser^179^), located in the first turn of αC cleft, and the sequence C^164^I^165^M^166^ associated with the characteristic “Met-turn”. These structural features are typical of the metzincin superfamily of metalloproteinases [14,18,19,20,21,23].

As depicted in Figure 2, primary structures of P-I SVMPs identified in our laboratory: leuc-a [29,45], mut-II [30], atroxlysin-I (atr-I, [28]), and barnettlysin-I (bar-I, [34]), align with other homologous P-I SVMPs, and show their high similarity. Nevertheless, despite their high sequence homology, some SVMPs induce hemorrhage, while others are (almost) inactive, and fail to cause any bleeding. This functional difference is probably related to the structural determinants in the MP domain [19,20,21,23,28,29,30,31]. In connection with this, Wallnoefer and collegues [46] have investigated the protein–protein interfaces of four P-I SVMPs, including: hemorrhagic (BaP1 and acutolysin A) and non-hemorrhagic (leuc-a and H2-proteinase ones). The P-I SVMPs hydrolyze basement membrane (BM) proteins in good correlation with their ability to bind them and to induce profuse bleeding in vivo. The authors applied computer simulations to obtain information about the backbone flexibility in certain surface regions/loops of these enzymes to carry out their damaging function. The findings indicated that the sequences of these four MPs mainly differ in the loop following the highly conserved active site, which surrounds the so-called Met-turn. For instance, the active hemorrhagic MPs (BaP1 and acutolysin A) both present a GSCSCGA/GKS (residues 154–163) before the Met-turn, whereas, the inactive (leuc-a and H2-proteinase) do not show any identical residues in this section, besides the two conserved Cys residues. This added further evidence to the hypothesis that flexibility might play a role in distinguishing between active and inactive enzymes. Thus, a certain combination of flexibility (residues 156–165) and rigidity of the neighboring loop C-terminal of the Met-turn (residues 167–176) provides an appropriate association domain for individual target protein [46,47]. However, despite intense investigation on this topic, detailed structural determinants of hemorrhagic activity have remained unclear, and no experimental data have been provided yet.

## 4. Action on Some Plasma and ECM Protein Substrates

Most of the relevant proteolytic enzymes that act on fibrin (Fb) and fibrinogen (Fbg) belong to one of two families: the metalloproteinases, and the serine proteinases. These proteinases can lead to defibrinogenation of blood, lysis of fibrin clots, and a consequent decrease in blood viscosity. Therefore, they can be regarded as true anticoagulants. The majority of fibrin(ogen)olytic enzymes are metalloproteinases which selectively cleave the α-chains of fibrin(ogen) to a ~44 kDa fragment and thereby are termed as α-fibrinogenases [4,28,29,57]. However, generalizations about chains specificity are not always applicable, since the other chains of fibrinogen can be substantially degraded over time. These P-I SVMPs are direct-acting fibrinolytics, as they are not reliant on components in the blood for activity. Like plasmin, the prototype of direct-acting fibrinolytic enzyme, a number of P-I SVMPs may represent an attractive therapeutic option in thrombolysis to allow reperfusion of ischemic tissue [58,59,60,61,62,63]. Among other P-I SVMPs described in the literature, alfimeprase, the recombinant form of fibrolase that first was isolated from the venom of the southern copperhead snake (*Agkistrodon contortrix contortrix*) [4,57,63], made the best progress, and has been investigated as a potential and safe thrombolytic agent, using in vitro, and also, many animal thrombosis models [57,63,64,65,66]. Alfimeprase proved to have clinical potential for drug development as a direct thrombolytic compound, however, the enzyme failed to successfully complete phase 3 clinical trials. Thus, Nuvelo (San Carlos, CA, USA) has discontinued further clinical development (for details, see [57]). A possible reason for suboptimal performance of alfimeprase in clinical use is its inability to bind to fibrin, thereby failing to reach a critical concentration of proteolytic activity locally at the thrombus [57]. Based on previous studies with fibrolase [67,68], Prof. Markland and his group at University of Southern California (Los Angeles, CA, USA), have not given up hope for alfimeprase and have constructed a chimeric compound possessing both thrombolytic and antiplatelet properties, and now the potential of this enzyme with bifunctional activity could be investigated in animal models of arterial thrombosis [57].

In studies of the vascular system, blood coagulation, fibrinolysis, and platelet function, snake venom proteins have been crucial in elucidating the complex physiological mechanisms which rule the vascular system, the coagulation cascade, and platelet functions. Moreover, they have been instrumental in elucidating the structure–function relationships of human clotting factors and platelet glycoproteins, because of their potency, selectivity, and high biological efficacy [65,66,67,68,69,70]. Notably, in the past fifteen years, the field has advanced due to the continued development of new or alternative agents that provide greater hemostatic safety and thrombolytic efficacy, as well as the identification of risk factors for arterial and venous thrombosis [71,72,73]. In this context, all the thrombolytic agents in current therapeutic use for deep vein thrombosis (DVT) e.g., the variants of tissue type plasminogen activator (tPA) are plasminogen activators (PAs). They efficiently dissolve thrombi, but adversely carry the unavoidable risk of bleeding complications [74,75].

To elucidate the molecular mode of action of four P-I SVMPs, identified in our laboratory: bar-I [34], leuc-a [29,45], mut-II [30], and atr-I [28], we have characterized the peptide bond specificity (Table A1). This is essential to understand the active site preference of the proteinases in correlation to their proteolytic and hemorrhagic activity. Moreover, this offers the opportunity to design peptide substrates and proteinase inhibitors. In addition to oxidized insulin B chain, which was used as the standard to compare peptide bond specificity among SVMPs, we also employed the designed peptides ^406^RREYHTEKLVSKGD^420^ and ^693^GHARLVHVEEPH^704^ as model substrates [34]. These sequences mimic the Aα-chain of Fbg and the “bait” region of α2-macroglobulin (α2-M), respectively. The results suggest that the SVMP-mediated cleavage is directed to an X–Leu bond, where X is a small residue at the P1 position, and a bulky hydrophobic residue at P1’, with a clear preference for leucine residue. An interesting study was carried out analyzing a plasma based, proteome-derived peptide library as substrate with mass spectrometry, to investigate the peptide bond specificity of three P-I SVMPs: atrolysin C (*C. atrox*, [75]), BaP1 [53], leuc-a [29], and a P-III bothropasin (*B. jararaca*, [76]). This study revealed the consensus sequence, ETAL–LLLD, that was similar to the other P-I enzymes, except of the acidic aspartate residue at the P4’ position for leuc-a [77,78]. These interesting differences in the peptide bond specificities at the other P and P’ sites, may imply functional differences between these proteases. For instance, the P-I enzymes showed preferences across the full P4 to P4’ range, whereas the P-III bothropasin exhibited narrow preferences across the sites, in accordance with earlier studies related with P-III SVMPs [78]. Furthermore, in the case of the non-hemorrhagic leuc-a, the preference for the acidic residue (Asp) in the P4’ site may have had a negative effect in inducing hemorrhage by this proteinase [77]. This finding merits further investigation for understanding the mechanism by which SVMPs induce hemorrhage. Moreover, the manner by which these enzymes act on various plasma and ECM proteins, including Fbg, FN, LM, fibrin, and collagen I and IV, were also performed. It is known that disruption of capillaries is the result of proteolytic degradation of key BM and ECM components, allowing for the escape of blood components into the stroma, and thus, producing local hemorrhage [22,28,36,37]. A comparative study of two P-I SVMPs: BaP1 (hemorrhagic) and leuc-a (non-hemorrhagic), provided insights into the putative mechanism of bleeding produced by SVMPs [36]. Both enzymes showed differences to degrade BM and associated ECM protein substrates, in vivo, mainly type IV collagen that is degraded by BaP1. To support these findings, further in vivo studies indicated that hydrolysis of type IV collagen by SVMPs, mainly P-II and P-III classes, is crucial in destabilizing microvessel structures and causing hemorrhage [79].

## 5. Antiplatelet Properties of P-I SVMPs

**Figure 4:**
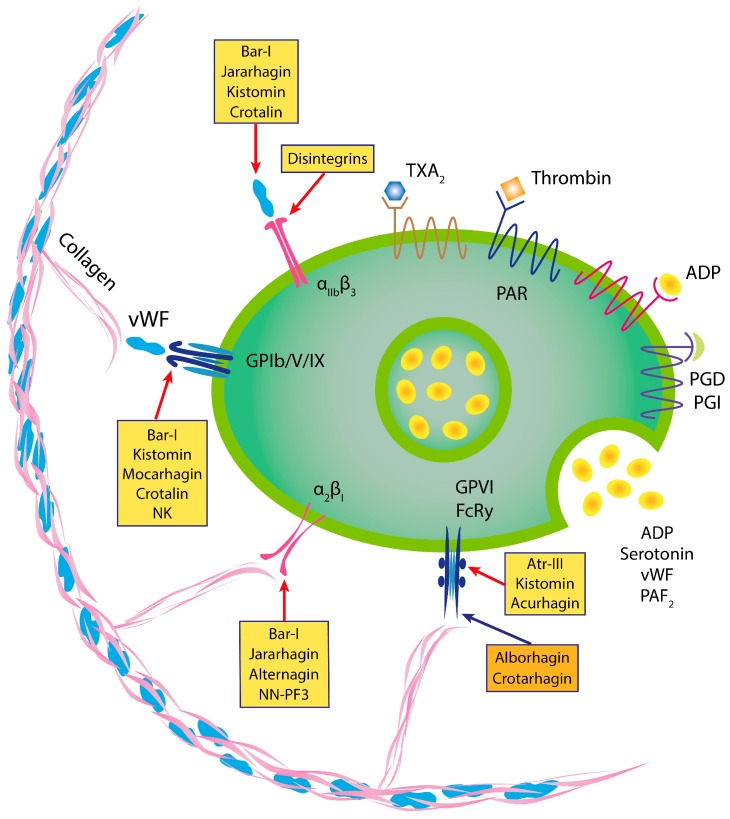
Schematic representation of some SVMPs and their effects on platelet function. Induction or inhibition of platelet aggregation by these proteinases are indicated in blue or red arrows, respectively. The inhibitory activity of disintegrins, the antagonists of αIIbβ3 integrin are indicated in red arrow. The ligands for various receptors are shown. PAR, protease activated receptor; TXA2, thromboxane A_2_; PGD, prostaglandin D; PGI, prostaglandin I. Modified from [88], copyright 2016 MDPI.

Blood platelets play a crucial role in hemostasis, and in the development of arterial thrombosis and of cardiovascular diseases. In response to vascular injury they rapidly adhere to exposed subendothelial matrix proteins, mainly von Willebrand factor (vWF) and collagen. As a result, adherent platelets are activated, spread, and release the content of storage vesicles [80,81]. More importantly, the main targets for antithrombotic drugs development are platelets and coagulation proteins [82,83]. According to current knowledge, the pathophysiology of arterial thrombosis differs from that of venous thrombosis as a consequence of higher shear forces in the arterial branch of the circulation, which require especially vWF and its shear-force-dependent conformational change for platelet adhesion [84]. Therefore, arterial thrombosis is treated with drugs that target platelets, while venous thrombosis is treated with drugs that target compounds of the coagulation cascade [71,72,73]. On the other hand, it has become clear that platelet function can be inhibited to reduce thrombotic tendencies by blocking either surface receptors, key cytoplasmic enzymes, e.g., cyclooxygenase or signaling proteins, including kinases or phosphatases [85,86]. As shown in Figure 4, there are a number of SVMPs that affect platelet aggregation. Notably, the glycoproteins (GPs), GPIb-IX-V and GPVI, receptors for vWF and collagen, respectively, bind to their respective ligands in different ways [85,86]. While at low physiological shear conditions, GPVI binds collagen exposed within the damaged blood vessel walls, and GPIb-IX-V binds vWF which has undergone conformational changes after exposure to high shear rates in arterioles and stenotic arteries [82,86,87]. Notably, only a small number of P-I SVMPs with direct-acting fibrinolytic activity inhibit platelet aggregation. Therefore, it is arguable whether fibrinogen degradation products generated by α-fibrinogenases play a role in inhibiting platelet aggregation [88].

An α-fibrinogenase, termed kistomin was indentified in the venom of *Calloselasma rhodostoma* (formerly *Agkistrodon rhodostoma*) during the 1990s. The enzyme inhibited platelet aggregation induced by low concentrations of thrombin (≥0.2 U/mL). Moreover, it attenuated cytosolic calcium rise and blocked thromboxane B2 formation in platelets stimulated by thrombin (0.1 U/mL). Importantly, the enzyme inhibited ristocetin-induced platelet aggregation in the presence of vWF. Thus, kistomin was the first P-I SVMP that exhibited anti-thrombotic effects [89]. Further investigation demonstrated that kistomin specifically inhibited vWF-induced platelet aggregation through binding and cleavage of platelet GPIbα and vWF [90]. Incubation of human platelets with kistomin resulted in a selective cleavage of platelet membrane glycoprotein GPIbα, with the release of ~45 and 130 kDa soluble fragments [90]. In addition, tail-bleeding time was prolonged in mice which had been injected with kistomin intravenously. The platelet receptor GPIb-IX-V complex plays a dominant role in the first steps of platelet adhesion under high shear stress conditions and arterial thrombus formation. Since GPIb–vWF interaction is very significant for hemostasis/thrombosis, the modulation of GPIbα–vWF interactions during thrombotic events could be beneficial [91,92]. Another P-I metalloproteinase, named crotalin, was purified from *Crotalus atrox* venom by the group of Prof. Huang [93]. Crotalin showed potent antithrombotic effect in vivo by cleaving vWF and GPIb, as shown by Western blotting and flow cytometry. Moreover, the author stated that crotalin, due to its multiple actions, may be a useful tool for investigating the interactions among vWF, ECM proteins, and GPIb-IX-V in static and flow conditions [93].

Based on the preliminary observations that kistomin and crotalin inhibited platelet aggregation induced by ristocetin, which promote vWF binding to GPIb, and taking into consideration that platelet dysfunction is responsible for increased patient morbidity and mortality, platelets represent the major target for therapeutic intervention [82,83,94]. Moreover, as thrombus formation is primarily stabilized by platelets and fibrin [73], we investigated a recently purified P-I metalloproteinase bar-I [34], and focused not only on the interaction of GPIb-IX-V complex with its major ligand, vWF, but also on other platelet surface ligands, such as fibrin. Its antithrombotic effect by targeting this receptor and potential platelet ligands, as well as its potential benefits, are worth discussing. Bar-I (23 kDa) was characterized as a direct-acting fibrinolytic enzyme which does not require the conversion of the zymogen plasminogen to the active form plasmin. Its amino acid sequence shows high sequence similarity with homologous P-I SVMPs (Figure 2). Furthermore, bar-I hydrolyzed several plasma and extracellular matrix (ECM) proteins in vitro [34,94]. Although SVMPs have similar proteolytic activity toward several substrates in vitro, bar-I is devoid of hemorrhagic activity in the mice skin model [34]. More importantly, the enzyme dose-dependently inhibited collagen- and plasma vWF-induced platelet aggregation. This effect was inhibited by treatment bar-I with EDTA, suggesting that the effect of bar-I on platelet activation is due to its enzymatic activity. Furthermore, vWF-induced platelet activation was more efficiently inhibited than collagen-induced platelet activation by this enzyme with (IC_50_) values of 1.4 and 3.2 μM, respectively. Interesting, studies in which platelets were pretreated with bar-I revealed that the vWF-receptor, GPIb-IX-V complex, is more susceptible to bar-I cleavage than the collagen binding receptor [34]. In addition to the cleavage of the multimeric adhesive protein vWF and its receptor GPIb, bar-I also cleaves the collagen receptor α2β1 integrin, albeit at a slower rate [34]. The essential interaction of GPIb-IX-V complex with vWF for normal hemostasis is well documented by the severe bleeding disorders as a consequence of the lack of either the receptor (Bernard–Soulier syndrome) or the ligand (von Willebrand disease). Thus, interactions of platelets via their receptors GPIb-IX-V and α2β1 integrin with vWF and collagen, respectively, indicates early events in platelet activation, especially under high shear rates of the arterial flow [73,83,95]. Furthermore, we have tested bar-I’s antithrombotic effect in vivo, with a tail bleeding assay using mice CF2 strain (18–20 g). When bar-I at doses of 2.5, 5 and 7.5 μg were injected intravenously into mice, the tail bleeding time was not altered (83 ± 4 s, *n* = 5) in comparison with saline (negative control, 78 ± 5 s, *n* = 5), (Sanchez et al., unpublished results). Similar data have been reported for batroxase, a P-I SVMP from *B. atrox* [96]. Moreover, bar-I is an analog of mut-II that disrupts formed thrombi through the hydrolysis of fibrin, rather than by plasminogen activation. We are now much interested in examining the hypothesis that vascular recanalization in the absence of plasmin generation results in improved thrombolysis without inducing bleeding side effects.

## 6. Biochemical Advantages of P-I SVMPs in Comparison to Plasminogen Activators (PAs)

One approach to treat thrombosis is to infuse thrombolytic agents to dissolve the blood clots and to restore tissue perfusion and oxygenation. This is ultimately accomplished by the serine proteinase plasmin, which is derived from its zymogen plasminogen in a reaction catalyzed by plasminogen activators (PAs), e.g., tissue type plasminogen activator (tPA), urokinase type-PA (u-PA) and staphylokinase. PAs effectively dissolve relatively small clots which occur in the coronary artery of patients with acute myocardial infarction [59,74,97,98]. Importantly, long retracted clots which are found in peripheral arterial occlusion (PAO) and deep-vein thrombosis (DVT), often lack plasminogen. Therefore, plasmin should be more efficacious to treat this kind of thrombus successfully [97,98,99,100,101].

**Figure 5:**
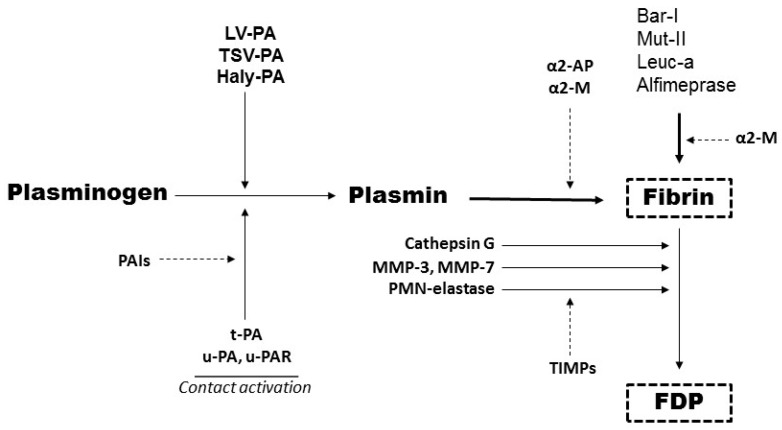
Schematic representation of the plasminogen/fibrinolytic system. Local effects of plasminogen activators (PAs) (physiological and snake venom PAs) and direct-acting fibrinolytic agents. PAs convert plasminogen to active enzyme plasmin, which degrades fibrin. In parallel using fibrin as substrate, direct-acting P-I SVMPs or endogenous plasmin proteolytically degrade fibrin and dissolve the fibrin clot. Inhibition (indicated by dotted lines) occurs either (i) at the level of PAs by plasminogen activator inhibitor (mainly by PAI-1 and PAI-2) or (ii) by plasmin inhibitors (by α2-antiplasmin and α2-macroglobulin). Matrix metalloproteinases (MMPs) degrade fibrin into smaller fragments, termed fibrin degradation products (FDP), and are inhibited by tissue inhibitor of MMPs (TIMPs). Snake venom serine proteinases, PAs (TSV-PA, LV-PA and Haly-PA [62]) and the direct-acting fibrinolytic metalloproteinases (bar-I, leuc-a, alfimeprase and mut-II), are shown at their site of inhibition.

Hemorrhage is a common complication of all the PAs, and is observed with any of the multiple agents and therapeutic indications [101]. An important conceptual framework of fibrinolytic activation and inhibition have been reported, and provided a foundation for understanding the mechanism of action of PAs and plasmin in a thrombus milieu [100,101,102]. A notable issue of direct-acting thrombolytics, of which plasmin is the prototype, has been well documented in in vitro and in vivo models, such as the use of catheter-delivered plasmin for treatment of DVT and PAO. As reported, plasmin should induce safe and efficacious thrombolysis at the thrombus site, whereas circulating enzyme is rapidly neutralized by α2-antiplasmin and by α2-macroglobulin (α2-M). This regime avoids bleeding complications, for details see [73,101,102]. Direct thrombolytic agents under investigation can be grouped into two categories: (a) plasmin and its derivatives: mini-plasmin, micro-plasmin, and delta-plasmin [74]; and (b) fibrinolytic SVMPs [63,99,100]. Figure 5 shows a schematic representation of the plasminogen/fibrinolytic system, including a number of snake venom proteins. Although plasmin is involved in other normal and pathological conditions, such as cell migration, inflammation and tissue remodeling, the most important function of plasmin is intravascular thrombolysis [58,59,98].

Fibrinolytic activity in viperid snake venoms was described for the first time in 1956 [60], when several pit viper venoms of *Agkistrodon*, *Bothrops*, and *Crotalus* genus were examined. Furthermore, the fibrinolytic activity in the venom of *A. contortrix contortrix* was reported to act directly on fibrin and independently of activation of the endogenous fibrinolytic system [60,61]. These findings supported a significant clinical potential of the direct thrombolytic agents to degrade fibrin without requiring an intermediate step of plasminogen activation. Therefore, alfimeprase, the recombinant fibrolase from *A. c. contortrix* venom, was used in clinical trials. In contrast to plasmin, this P-I SVMP does not bind directly to fibrin. In addition, the systemic effects of venom fibrinolytic enzymes are inhibited by α2-M, which is the last line of defense against exogenous proteolytic enzymes. As direct-acting thrombolytic P-I SVMPs are not inhibited by the normal blood serine proteinase inhibitors (serpins), they may serve as templates for the development of alternative thrombolytic compounds, and have received special attention due to their possible therapeutic role for dissolution of blood clots [62,63,64,65,66,67,68,69,70]. These enzymes act on fibrin and Fbg, leading to defibrinogenation of blood, lysis of fibrin clots, and a consequent decrease in blood viscosity [69]. They may be classified as being either α or β chain fibrin(ogen)ases. Due to their broad spectrum of proteolytic activity leading to fibrin(ogen) digestion, they can be regarded as true anticoagulants, and are metalloproteinases or serine proteinases [29,34,69]. Also, it was observed that the level of fibrinolytic effect varies widely between the P-I SVMPs of different species within a particular genus [60,61,62,63,65]. Therefore, it was suggested to use a properly purified enzyme(s) from snake venoms as fibrin clot-lysing agent for clinical applications. In the early 1990s, we reported the isolation and complete amino acid sequence of a P-I class metalloproteinase, termed lachesis hemorrhagic factor II (LHF-II), from bushmaster (*Lachesis muta muta*) snake venom [30]. Additionally, we had elucidated that traces of hemorrhagic effect of LHF-II were due to the presence of a minor contaminant by the P-III metalloproteinase named mutalysin-I (mut-I). Therefore, the proteinase was renamed to mutalysin-II (mut-II). Furthermore, its pharmacological properties have been reevaluated, and these reports had indicated that mut-II does not elicit any hemorrhagic response in mice or rabbit [32]. More importantly, we have evaluated by intravital microscopy, the effects of mut-II on the recanalization of microvessels after thrombus induction in the ear of hairless mice. At the doses used (50 μg, 2.0 mg/kg, iv), thrombolytic efficacy was achieved in all animals (*n* = 5) after approximately 12 min, followed by recanalization. A control group (*n* = 5) that received u-PA (250 U/mouse, iv), showed blood flow restoration within the same interval, 12 min. In addition, under the experimental conditions, mut-II does not alter hemostasis or cause bleeding events, as confirmed by histopathology [32]. Based on these data, we have also initiated in vivo studies to assess the thrombolytic potential of a recently isolated bar-I by using intravital microscopy in comparison with recombinant t-PA. This direct-acting fibrinolytic enzyme dissolves fibrin clots in vitro, and also inhibits collagen- and plasma vWF induced platelet aggregation by cleaving not only the vWF and its receptor GPIb, but also the collagen receptor α2β1 integrin. Although the current thrombolytic agents have proved to be of clinical benefit, the failure to rapidly restore reperfusion in some patients, and the continuous risk of bleeding of all PAs, are still setbacks which have to be improved in order to introduce them in the routine of clinical therapy [72,82,83,100]. Therefore, continued development of safer and more efficient thrombolytic agents, in combination with more effective antiplatelet approaches, are the future goals in this research field.

## 7. Conclusions

Fibrin clot-based vascular occlusion, a life-threatening disorder, has to be treated immediately by dissolving the fibrin clot in the vessel which impairs the blood flow. Treatment with recombinant plasmin or with plasminogen activator is usually the choice of means in the hospital. As an alternative, recombinantly produced P-I SVMPs are investigated. Their extremely high fibrinolytic activity gives them an advantage over current fibrin clot-dissolving agents. However, substrate specificity of such P-I SVMP should be mainly limited to fibrin. Any cleavage or degradation of the blood vessel wall, especially of the basement membrane subjacent to the endothelial cells, must be absent to avoid blood leakage and hemorrhages. Whereas several P-I SVMPs cleave vessel wall components, some non-hemorrhagic P-I SVMPs have been identified. The structural comparison between both groups may reveal characteristics for non-hemorrhagic P-I SVMPs to accelerate the search for such fibrinolytic, non-hemorrhagic P-I SVMPs in the biodiversity of snake venoms. Thereby, lead-structures can be obtained for the design of novel fibrinolytic, non-hemorrhagic proteinases. Some of the recent non-hemorrhagic P-I SVMPs also cleave adhesion receptors on platelets, such as vWF-receptor. Cleavage of those “off-targets” would be tolerable, if not even beneficial, as such a proteinase would have, in addition to their fibrinolytic activity, antithrombotic functions by preventing platelets from adhesion and thrombus formation. Moreover, platelets support leukocyte extravasation. Although the molecular mechanism is not fully understood, neutrophils seem to interact with platelets and use their adhesive potential, likely via their adhesion receptors, GPIb and αIIbβ3, to attach to the vessel wall, especially under higher shear rates and at atheroslerotic lesions [103]. Similarly, blood-borne tumor cells during hematogenic dissemination also interact with platelets and subvert their adhesive potential to the vessel wall, likely also mediated via the vWF-receptor and different integrins [104]. Hence, it is worthwhile to think whether non-hemorrhagic snake venom proteinases which cleave the adhesion receptors on platelets might be useful in reducing platelet-supported extravasation of leukocyte, or disseminating blood-borne tumor cells. This could be a strategy to reduce formation of atherosclerotic plaques or metastasis. Another criterion for the use of fibrinolytic, non-hemorrhagic P-I SVMPs is the restriction of the fibrinolytic activity to the thrombus site and to prevent potential adverse systemic effects. A long way ahead, but the goal of utilizing fibrinolytic, non-hemorrhagic P-I SVMPs in clearing thrombotic occlusions or inhibiting platelet-assisted cell extravasation is promising.

## Figures and Tables

**Table 1:** Three dimensional structures of P-I class SVMPs deposited in the PDB and their main biological activities.

SVMP	Source	Activities	PDB ID	Year	Reference
Adamalysin II	*C.* adamanteus	non-hemorrhagic	1LAG	1993	[21]
Atrolysin C	*C. atrox*	hemorrhagic	1ATL, 1HTD	1994	[48]
H2 proteinase	*T. Flavoviridis*	non-hemorrhagic	1WNI	1996	[49]
Acutolysin A	*A. Acutus*	hemorrhagic	1BSW,1BUD	1998	[50]
Acutolysin C	*A. Acutus*	hemorrhagic	1QUA	1999	[51]
TM-3	*T. Mucrosquamatus*	fibrinogenolytic	1KUF, 1KUI	2002	[52]
BaP1	*B. asper*	hemorrhagic	1ND1	2003	[53]
FII	*A. acutus*	non-hemorrhagic	1YP1	2005	[54]
BmooMPα-I	*B. moogeni*	non-hemorrhagic	3GBO	2010	[55]
TM-1	*T. mucrosquamatus*	fibrinogenolytic	4J4M	2013	[56]
Leuc-a	*B. leucurus*	non-hemorrhagic	4Q1L	2015. unpublished	

## Appendix A

**Table A1:** Cleavage sites of three synthetic substrates by some P-I SVMPs.

Proteinase	Bonds Cleaved	Reference
Oxidized Insulin B chain		
*leuc-a*	Ala_14_-Leu_15_, Tyr_16_-Leu_17_	[29]
*atr-I*	Ala_14_-Leu_15_, Tyr_16_-Leu_17_	[28]
*BaP1*	Ala_14_-Leu_15_, Tyr_16_-Leu_17_	[36]
*mut-II*	His_5_-Leu_6_, His_11_-Leu_11_, Ala_14_-Leu_15_, Phe_24_-Phe_25_	[78]
Human α2-M (bait region)		
*leuc-a*	Arg_696_-Leu_697_	[105]
*atr-I*	Arg_696_-Leu_697_	[unpublished]
*mut-II*	Arg_696_-Leu_697_	[unpublished]
*bar-I*	Arg_696_-Leu_697_	[34]
Human fibrinogen Aα-chain		
*leuc-a*	Lys_413_-Leu_414_	[105]
*atr-I*	Lys_413_-Leu_414_	[unpublished]
*mut-II*	Lys_413_-Leu_414_	[unpublished]
*bar-I*	Lys_413_-Leu_414_	[34]
